# A new arylbenzofuran derivative functions as an anti-tumour agent by inducing DNA damage and inhibiting PARP activity

**DOI:** 10.1038/srep10893

**Published:** 2015-06-04

**Authors:** Hongbo Chen, Xiaobin Zeng, Chunmei Gao, Pinghong Ming, Jianping Zhang, Caiping Guo, Lanzhen Zhou, Yin Lu, Lijun Wang, Laiqiang Huang, Xiangjiu He, Lin Mei

**Affiliations:** 1 The Shenzhen Key Lab of Gene and Antibody Therapy, The Ministry-Province Jointly Constructed Base for State Key Lab-Shenzhen Key Laboratory of Chemical Biology, and Division of Life and Health Sciences, Tsinghua University Shenzhen Graduate School, Shenzhen 518055, China; School of Life Sciences, Tsinghua University, Beijing 100084, China; 2 Guangdong Key Laboratory for Research and Development of Natural Drugs, Guangdong Medical College, Zhanjiang 524023, Guangdong, China; 3 Clinical laboratory, Zhuhai People’s hospital, Zhuhai 519000, China; 4 Shenzhen Weiguang Biological Products Co., Ltd, Shenzhen 518107, China; 5 Drug Discovery and Design Center (DDDC), Shanghai Institute of Materia Medica, Shanghai 201203, China; 6 College of Pharmacy, Guangdong Pharmaceutical University, Guangzhou 510006, China

## Abstract

We previously reported that 7-hydroxy-5, 4’-dimethoxy-2-arylbenzofuran (HDAB) purified from *Livistona chinensis* is a key active agent. The present study investigated the function and molecular mechanism of HDAB. HDAB treatment of cervical cancer cells resulted in S phase arrest and apoptosis, together with cyclin A2 and CDK2 upregulation. Cyclin A2 siRNA and a CDK inhibitor efficiently relieved S phase arrest but increased the apoptosis rate. Mechanistic studies revealed that HDAB treatment significantly increased DNA strand breaks in an alkaline comet assay and induced ATM, CHK1, CHK2 and H2A.X phosphorylation. Wortmannin (a broad inhibitor of PIKKs) and CGK733 (a specific ATM inhibitor), but not LY294002 (a phosphatidylinositol 3-kinase inhibitor) or NU7026 (a DNA-PK specific inhibitor), prevented H2A.X phosphorylation and γH2A.X-positive foci formation in the nuclei, reversed S phase arrest and promoted the HDAB-induced apoptosis, suggesting that HDAB is a DNA damaging agent that can activate the ATM-dependent DNA repair response, thereby contributing to cell cycle arrest. In addition, molecular docking and *in vitro* activity assays revealed that HDAB can correctly dock into the hydrophobic pocket of PARP-1 and suppress PARP-1 ADP-ribosylation activity. Thus, the results indicated that HDAB can function as an anti-cancer agent by inducing DNA damage and inhibiting PARP activity.

Cervical cancer is one of the most common malignant tumours worldwide and remains a leading cause of cancer-related death among women in developing countries^1^. The causal relation between genital human papillomavirus (HPV) infection and cervical cancer is well established. Among all the types of HPV, types 16 and 18 are the most dangerous and are responsible for approximately 70 percent of all cases of cervical cancer^2^,^3^,^4^. Recently, the Food and Drug Administration (FDA) approved two HPV vaccines (Gardasil® and Cervarix®) directed against HPV types 16 and 18. The use of these vaccines has been shown to effectively prevent cervical cancer by protecting women against infection with HPV types 16 and 18^5^,^6^,^7^. However, these vaccines do not have therapeutic effects against pre-existing HPV infections and do not prevent the progression of HPV-associated lesions. Unfortunately, the incidence rate of cervical cancer is expected to continue increasing in the next decades^8^. Current therapeutic regimens for cervical cancer include surgical removal, radiotherapy and chemotherapy. However, the common combination therapy has a maximum response rate of only 30%, and patients with cervical cancer have a median overall survival of less than 17 months due to the lack of an effective chemotherapy regimen^9^. Therefore, novel effective chemotherapy drugs for cervical cancer are urgently required.

*Livistona*, a genus of approximately 36 species of palms, is widely distributed in southern and southeastern Asia, Australasia, and the Horn of Africa^10^. Their fruits have been used for analgesic and haemostatic purposes in traditional Chinese medicine. In recent years, *Livistona* extracts have been shown to have potent anti-proliferation activity against multiple tumour cells, including human myeloid leukaemia cells, gastric cancer cells, cervical cancer cells, liver cancer cells, melanoma cells, colon cancer cells, and bladder cancer cells^11^,^12^. Our lab isolated and identified a new compound, 7-hydroxy-5,4’-dimethoxy-2-arylbenzofuran (HDAB), from the fruits of *L. chinensis* (Fig. 1)^13^. In our preliminary study, HDAB significantly inhibited the growth of a number of malignant cell lines, particularly cervical cancer cell lines (Table 1). In the present study, the activity of HDAB and the mechanisms by which it exerts its anti-proliferative effects were further investigated.

## Results

### Effects of HDAB on the growth and proliferation of cervical cancer cells

To examine the biological effects of HDAB, various cancer cell lines were treated with several concentrations of HDAB (0 μM, 4.6 μM, 9.2 μM, 18.4 μM, 36.8 μM, or 73.6 μM) for 24 h and 48 h, and cell viability was assayed by the MTT method. The 50% inhibitory concentrations (IC_50_) of HDAB against the human tumour cell lines are shown in Table 1. The results suggested that HDAB significantly inhibited the growth and proliferation of all of the selected tumour cell lines. Based on these results, we selected the HeLa (HPV18-positive) and CaSki (HPV16-positive) cell lines to investigate the anti-tumour effects and mechanisms of action of HDAB.

**Figure 2:**
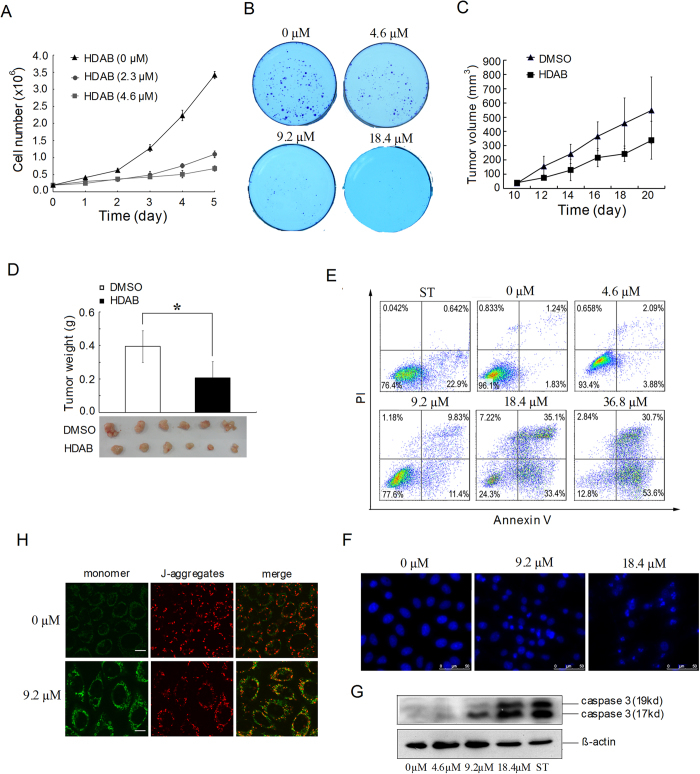
Effects of HDAB on the growth and apoptosis of cervical cancer cells. **(A)** HeLa cells were treated with the indicated concentrations of HDAB and cell number was calculated. The data are presented as the mean ±SD **(B)** Colony formation ability of HeLa cells treated with the indicated concentrations of HDAB in soft agar and imaged on day 10. (**C**) The growth curves of subcutaneous tumours in mice treated with HDAB or DMSO. The data are presented as the mean ±SD (*n* = 6 in each group). (**D**) Weights of the tumours from mice sacrificed 20 days after treatment (* *p* < 0.05). (**E**) Apoptosis was analysed by annexin V/PI staining after 24 h of treatment with staurosporine (0.5 μM) or HDAB at the indicated concentrations; representative histograms are shown. (**F**) Nuclear morphology was analysed by fluorescence microscopy after DAPI staining of cells treated for 48 h with HDAB; representative images are shown (Scale bar, 50 μm). (**G**) Activated caspase-3 was detected by Western blotting after 24 h of treatment with staurosporine (0.5 μM) or HDAB at the indicated concentrations. (**H**) Mitochondrial membrane potential was detected by JC-1 staining after 24 h of treatment with HDAB; representative images are shown (Scale bar, 10 μm).

Cell proliferation assay showed that low concentration of HDAB significantly inhibited the proliferation of HeLa cells compared with non-treated cells (Fig. 2A). Anchorage-independent colony formation is an important signature of malignant cervical cancer cells that correlates strongly with tumourigenic, invasive and metastatic potentials. Figure 2B shows that the colony formation ability of HeLa cells was also significantly inhibited by HDAB in a dose-dependent manner. A similar result was obtained in CaSki cells (data not shown).

To evaluate the *in vivo* anti-cancer activity of HDAB, the growth inhibition of HeLa xenografts in nude mice was investigated. The administration of HDAB resulted in significant growth suppression of HeLa xenografts compared to the control groups. As shown in Fig. 2C, tumour growth in the HDAB-treated group was significantly slower than that in the DMSO-treated group. At the end of the experiment, the average tumour weight in the HDAB-treated group was significantly lower than that in the DMSO-treated group (Fig. 2D). No statistically significant differences in body weight were observed between the HDAB-treated and DMSO-treated mice (*p* > 0.05, data not shown), indicating a low general toxicity of HDAB.

### Effects of HDAB on cervical cancer cell apoptosis

We also investigated whether HDAB can induce cervical cancer cell apoptosis. As depicted in Fig. 2E, HDAB treatment resulted in a significant increase in the percentage of annexin V-positive cells in a dose-dependent manner. The DAPI staining data were consistent with the annexin V assay data. The ratio of cells with apoptotic nuclear morphology (fragmented nuclei and condensed chromatin) to the total number of cells significantly increased at 24 h after treatment with HDAB compared to that of DMSO treatment (Fig. 2F). Caspase-3 is a key effector in the process of apoptotic cell death. Figure 2G shows that the activated form of caspase-3 was markedly up-regulated in HDAB-treated cells compared to that in control cells. Decreased mitochondrial membrane potential (MMP) is also a marker of apoptosis^14^,^15^. As expected, confocal microscopy showed that most DMSO-treated HeLa cells had strong staining of J-aggregates (red) and weak staining of JC-1 monomers (green). Conversely, HDAB-treated cells exhibited increased JC-1 monomer staining (green) and concomitantly decreased J-aggregate staining (red), suggesting a low MMP (Fig. 2H).

### Increased expressions of cyclin A2 and CDK2 contributed to the HDAB-induced S phase arrest but not to cytotoxicity and apoptosis

**Figure 3:**
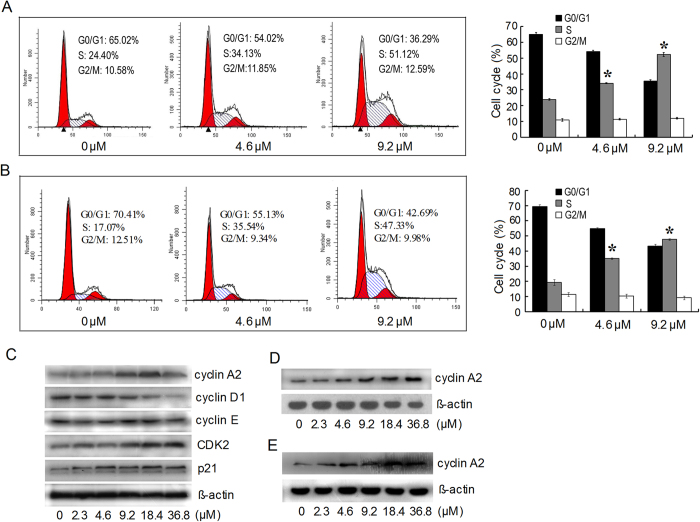
Effects of HDAB on the cell cycle in HeLa cells. (**A**) and **(B)** The cell cycle distributions of HeLa and CaSki were analysed by flow cytometry after 24 h of treatment with the indicated concentrations of HDAB, respectively; representative images are shown (left panel). Three independent experiments were performed; the data are presented as the mean ±SD (column, *p < 0.05). (**C**) Cell cycle-related proteins were detected by Western blotting after 24 h of treatment with the indicated concentrations of HDAB. (**D**) and **(E)** The expression of cyclin A2 in HeLa and CaSki cells treated with HDAB (9.2 μM) was detected at the indicated time points, respectively.

Based on the above results, we next assessed whether the observed HDAB-induced apoptosis and growth inhibition are accompanied by an effect on cell cycle progression. HeLa cells were significantly arrested in S phase when treated with either 4.6 μM or 9.2 μM HDAB for 24 h, with approximately 34% and 51% of the cells in S phase, respectively, compared to approximately 24% in the control group (Fig. 3A). A similar cell cycle distribution was observed in CaSki cells treated with HDAB (Fig. 3B). Faurthermore, the expression of cell cycle regulatory proteins was analysed by Western blotting. Consistent with S phase arrest, the expression of cyclin A2 and CDK2, which are known to promote S phase entry in mammals, significantly increased following exposure to HDAB in a dose- and time-dependent manner (Fig. 3C,D), whereas no obvious change was observed in cyclin E expression. The expression of cyclin D1, which is primarily expressed in early G0/G1, obviously decreased (Fig. 3C). An obvious increase of cyclin A2 expression level in CaSki cells was also observed (Fig. 3E).

**Figure 4:**
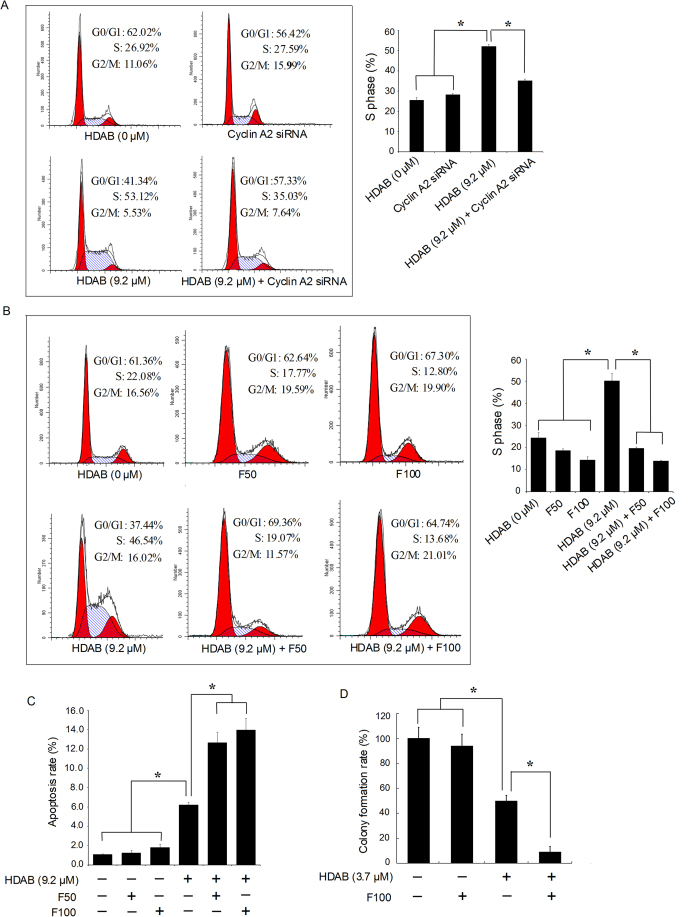
Cyclin A2 siRNA and flavopiridol reversed the HDAB-induced S phase arrest and enhanced apoptosis. (**A**) Cyclin A2-specific or control siRNAs were transfected into HeLa cells for 24 h, and then the cells were treated with or without 9.2 μM HDAB for an additional 24 h. The cell cycle distribution was determined by flow cytometry, and representative images are shown (left panel). Three independent experiments were performed; the S phase data are presented as the mean ±SD (column, *p < 0.05). (**B**) HeLa cells were pretreated with 50 nM (F50) or 100 nM (F100) flavopiridol for 2 h and then treated with or without 9.2 μM HDAB for an additional 24 h; the cell cycle distribution was analysed by flow cytometry. The data from three independent experiments are presented as the mean ±SD (*p < 0.05). (**C**) Cells were treated with or without 9.2 μM HDAB in the presence or absence of the indicated concentrations of flavopiridol, and apoptosis was examined (*p < 0.05). (**D**) HeLa cells were treated with or without 3.7 μM HDAB in the presence or absence of 100 nM flavopiridol, and colony formation was examined (*p < 0.05).

To determine the role of cyclin A2/CDK2 in the HDAB-induced growth arrest and apoptosis, two cyclin A2-specific siRNAs and the CDK2 inhibitor flavopiridol were administered to silence the expression of cyclin A2 or inhibit the kinase activity of CDK2. Western blot analysis showed that both siRNA1 and siRNA2 significantly inhibited the expression of cyclin A2 compared to control siRNA (data not shown). Therefore, siRNA1 was used in the subsequent experiments. As shown in Fig. 4A, the cell cycle distribution of the cells treated with only cyclin A2 siRNA did not showed significant difference compared with that of non-treated cells, while cyclin A2 knockdown effectively attenuated the HDAB-induced S phase arrest. Similarly, treatment with 50 nM and 100 nM flavopiridol abolished the HDAB-induced S phase arrest (Fig. 4B). However, unexpectedly, the attenuation of S phase arrest by cyclin A2 siRNA and flavopiridol further promoted the HDAB-induced apoptosis and colony formation inhibition in cervical cancer cells (Fig. 4C,D). Overall, these data suggested that the activation of cyclin A2/CDK2 by HDAB contributed to S phase arrest in cervical cancer cells. However, S phase arrest may be only a consequence of HDAB treatment and not the real cause of cytotoxicity and of apoptosis induction.

### HDAB treatment resulted in DNA damage and activated a DNA damage checkpoint response (DDR) and the DNA damage repair pathway

**Figure 5:**
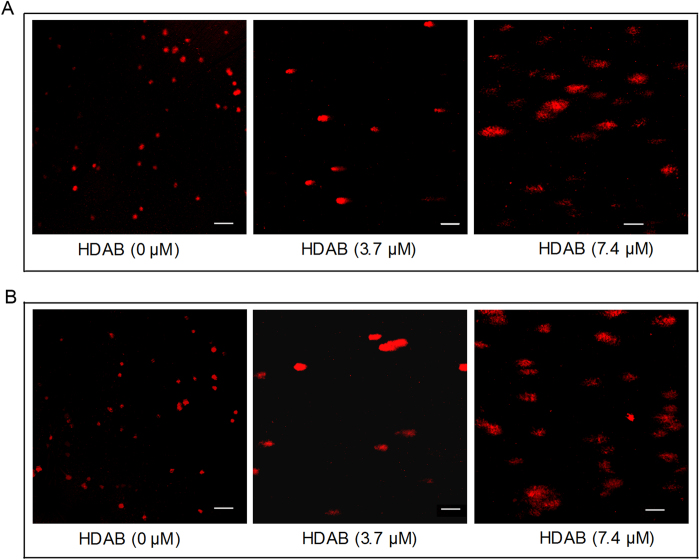
HDAB-induced DNA damage was detected by comet assays. **(A)** and **(B)** After treated with the indicated concentrations of HDAB for 24 h, DNA damage in HeLa and CaSki cells was detected by comet assays, respectively, and the slides were imaged using fluorescence microscopy (Scale bar, 50 μm).

Several studies have reported that DNA damage can result in S phase arrest and induce a DNA damage repair response^16^,^17^,^18^. When damaged DNA cannot be repaired, cells are likely to proceed to apoptosis. In the present study, a comet assay was used to determine whether HDAB treatment can result in DNA damage. As shown in Fig. 5A, untreated control HeLa cells had no detectable comet tails or had shorter comet tails, whereas cells treated with HDAB exhibited significant comet tail formations in a dose-dependent manner. Thus, this result suggested that HDAB treatment markedly induced DNA damage in HeLa cells. A similar result was observed in CaSki cells (Fig. 5B).

**Figure 6:**
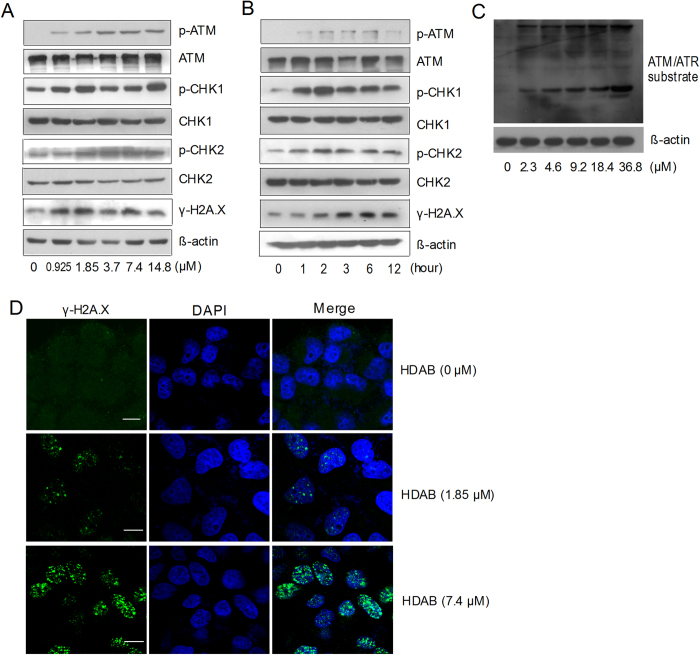
HDAB activated the ATM-dependent DNA repair/cell cycle checkpoint pathway. (**A**) HeLa cells were treated with the indicated concentrations of HDAB for 6 h; total cell lysates were prepared and subjected to SDS-PAGE, followed by Western blotting with the indicated antibodies. (**B**) HeLa cells were treated with 9.2 μM HDAB, and Western blotting was conducted at the indicated time points. (**C**) HeLa cells were treated with the indicated concentrations of HDAB for 12 h, and then total cell lysates were prepared and subjected to SDS-PAGE, followed by Western blotting with phospho-(Ser/Thr) ATM/ATR substrate antibody. (**D**) HeLa cells were treated with the indicated concentrations of HDAB for 12 h, and γH2A.X foci were observed and imaged as described in the **Methods** section (Scale bar, 10 μm).

The DNA damage response network is composed of a protein kinase cascade connecting the recognition of DNA damage to the activation of the DNA repair response, which involves cell cycle arrest, DNA repair and programmed cell death^19^,^20^,^21^,^22^. Thus, two sensors of DNA damage, ATM (ataxia-telangiectasia-mutated) and DNA-PK (DNA-dependent protein kinase), were detected by Western blotting. The expression of p-ATM significantly increased in a dose- and time-dependent manner after HDAB treatment (Fig. 6A,B), whereas p-DNA-PK levels did not change (data not shown). The HDAB-induced activation of ATM was further confirmed by Western blotting using phospho-(Ser/Thr) ATM/ATR substrate antibody. As shown in Fig. 6C, the phosphorylation of endogenous proteins containing the ATM/ATR substrate motif obviously increased in HeLa cells treated with HDAB for 12 h. Activated ATM phosphorylates two checkpoint kinases, CHK1 and CHK2, possibly leading to cell growth arrest. Next, we examined the phosphorylation levels of CHK1 and CHK2, which are the key downstream checkpoint substrates of ATM. As expected, HDAB treatment resulted in an obvious increase in the phosphorylation of CHK1 (Ser345) and CHK2 (Thr68) in a dose- and time-dependent manner (Fig. 6A,B). H2A.X is another phosphorylated target protein of ATM that is known to play an important role in the recruitment of repair factors to nuclear foci after DNA damage. As shown in (Fig. 6A,B), HDAB treatment induced an increase in phosphorylated H2A.X (Ser139) in a dose- and time-dependent manner. This observation was further supported by the immunocytochemical data showing that the frequency of γH2A.X foci per nucleus significantly increased after HDAB treatment (Fig. 6D).

### Inhibition of ATM activity abolished the HDAB-induced S phase arrest and promoted apoptosis

**Figure 7:**
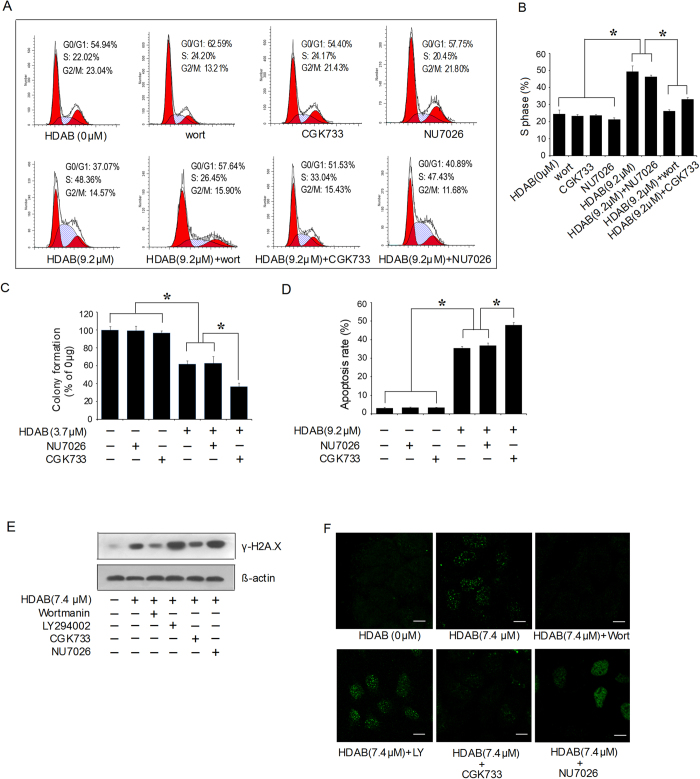
An ATM inhibitor reversed the HDAB-induced S phase arrest, inhibited DNA repair and enhanced apoptosis and colony formation inhibition. (**A**) HeLa cells were treated with or without HDAB (9.2 μM) in the presence or absence of wortmannin (100 nM), CGK733 (50 nM) and NU7026 (10 μM) as described in the Methods section; the cell cycle distribution was analysed, and representative histograms are shown. (**B**) Three independent experiments were performed; the S phase data are presented as the mean ±SD (column, *p < 0.05). (**C, D**) HeLa cells were treated with or without HDAB, CGK733 (50 nM) and NU7026 (10 μM) as described in the Methods section, and colony formation and apoptosis were detected. Three independent experiments were performed; the data are presented as the mean ±SD (column, *p < 0.05). (**E, F**) HeLa cells were treated with or without HDAB (9.2 μM) in the presence or absence of wortmannin (100 nM), LY294002 (5 μM), CGK733 (50 nM) and NU7026 (10 μM); phosphorylated H2A.X (Ser139) levels and γH2A.X foci were detected by Western blotting and confocal microscopy (Scale bar, 10 μm), respectively.

ATM and DNA-PK belong to the superfamily of phosphatidylinositol 3-kinase-related kinases (PIKKs), which share the PI-3-like kinase domain but do not function as lipid kinases^23^. To evaluate whether PIKKs are involved in the HDAB-induced S phase arrest, the cell cycle distribution was analysed after pre-treatment with wortmannin (a broad inhibitor of PIKKs), LY294002 (a lipid phosphatidylinositol 3-kinase inhibitor), CGK733 (a specific ATM inhibitor) or NU7026 (a DNA-PK-specific inhibitor)^24^,^25^,^26^. Figure 7A and B show that the cell cycle distribution of the cells treated with only wortmannin, CGK733 or NU7026 did not showed significant difference compared with that of non-treated cells, while both wortmannin and CGK733, but not NU7026 effectively reversed the HDAB-induced S phase arrest of HeLa cells. Furthermore, we evaluated whether ATM activation is involved in the HDAB-induced apoptosis and colony formation inhibition. As shown in Fig. 7C,D, the apoptotic rate and colony formation inhibition rate of the cells treated with only CGK733 or NU7026 did not showed significant difference compared with that of non-treated cells, while the ATM-specific inhibitor CGK733, but not the DNA-PK-specific inhibitor NU7026, promoted the HDAB-induced apoptosis and colony formation inhibition. Similarly, both wortmannin and CGK733, but not LY294002 and NU7026 effectively attenuated the HDAB-induced H2A.X phosphorylation and γH2A.X foci formation in the nucleus (Fig. 7E,F). These data suggested that ATM, but not DNA-PK, was responsible for the HDAB-induced S phase arrest and DNA damage repair. If the ATM repair pathway is inhibited, then cells initiate programmed cell death.

### HDAB bound to poly (ADP-ribose) polymerase (PARP) and inhibited its activity

**Figure 8:**
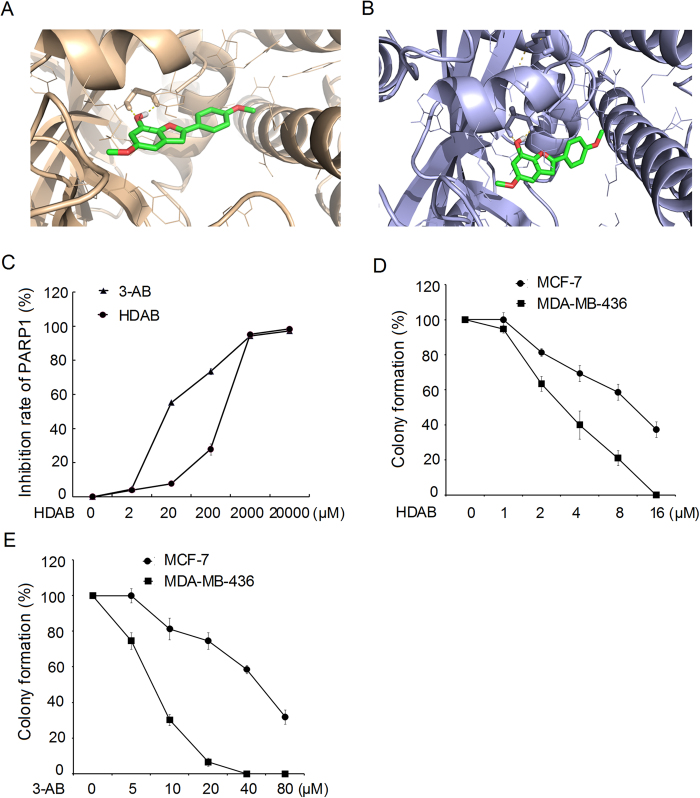
HDAB is a potential inhibitor of PARP-1 and PARP-2. (**A**, **B**) Computational modelling of HDAB binding to PARP-1 and PARP-2. (**C**) The inhibitory effect of HDAB on PARP-1 activity was measured using a PARP Assay Kit. The data from three independent experiments are expressed as relative inhibition rates; the inhibition rate in the control was set to 0. (**D**) Clonogenic survival assays of HDAB for MCF-7 and MDA-MB-436 human breast cancer cells. (**E**) Clonogenic survival assays of 3-AB for MCF-7 and MDA-MB-436 human breast cancer cells. Three independent experiments were performed and the data are presented as the mean ±SD. The colony formation of non-treated cells was set to 100.

The above results showed that HDAB is a DNA damage agent and can induce cell apoptosis. In addition, we presumed that HDAB may also be a potential PARP inhibitor by analysing its molecular structure. PARP-1 and PARP-2 play important roles in the DNA damage response. PARP-1/2 can bind to the DNA damage site and promote DNA repair by catalysing the placement of poly (ADP-ribose) on multiple DNA repair-related proteins^27^,^28^,^29^. To confirm this, a molecular docking assay was performed with the “induced-fit docking” program module of Schrodinger Suite 2010. The crystal structures of PARP-1 (3L3M) and PARP-2 (3KCZ) from the PDB (Protein Data Bank) were used for docking. Previous studies have reported that PARP-1 and PARP-2 have two active sites, pocket 1 (a nicotinamide binding site) and pocket 2 (a hydrophobic tail); pocket 1 has been used for screening PARP inhibitors^30^,^31^,^32^. As shown in Fig. 8A and B, HDAB is well anchored in the nicotinamide pockets of PARP-1 and PARP-2. In addition, HDAB can interact with PARP-1 pocket 1 *via* several hydrogen bond interactions, such as the interactions between the arylbenzofuran ring of HDAB and Gly202 and Ser243 of PARP1, which are similar to those of the 3L3M ligand. The docking results also showed that HDAB has pi-pi stacking interactions with Tyr246, Tyr245 and His201 of PARP-1.

The *in vitro* PARP-1 activity assay further confirmed the molecular docking results. Our data suggested that although the inhibitory effect of HDAB was less potent than that of a known PARP-1 inhibitor, 3-AB (IC_50_ = 51.9 μM), HDAB significantly inhibited PARP-1 enzyme activity, with an IC_50_ of 154.2 μM (Fig. 8C).

Many researches have reported that the breast cancer susceptibility gene (*BRCA*) -mutant cells were extremely sensitive to small molecule PARP inhibitors owing to their “synthetic lethality”^33^, ^34^, ^35^. We then evaluated the inhibition ability of HDAB to colony formation in MCF-7 (BCRA1 proficient breast cancer cell line) and MDA-MB-436 cells (BRCA1 deficient breast cancer cell line). Figure 8D demonstrated that HDAB induced reduced clonogenic survival of MDA-MB-436 cells compared to MCF-7 cells. As a positive control we tested 3-aminobenzamide (3-AB, a known PARP inhibitor) in this system and observed the similar result (Fig. 8E). These data indicated that HDAB can inhibit PARP activity and have a synthetic cytotoxicity with inactivation of BRCA function.

## Discussion

HDAB is a novel natural product isolated from *L. chinensis* that has potential antitumour activity against many types of cancer, particularly cervical cancer (Table 1). In the present study, the anti-cancer mechanism of action of HDAB in cervical cancer cells was further investigated. The current study showed that treating HeLa and CaSki cells with HDAB resulted in S phase arrest, cell growth inhibition and apoptosis (Figs. 2 and 3). Both cyclin A2 knockdown and CDK2 kinase inhibition effectively attenuated the HDAB-induced S phase arrest, suggesting that cyclin A2/CDK2-associated kinase activation is responsible for the S phase arrest (Fig. 4). Recently, increasing evidence has suggested that elevated expression of cyclin A and/or activation of CDK2 contribute to apoptosis and that inhibiting cyclin A/CDK2 activity can reverse drug-induced S phase arrest and apoptotic cell death; these data provide strong evidence that the activation of cyclin A/CDK2 is an important mediator of drug-induced S phase arrest and apoptosis^36^,^37^,^38^,^39^,^40^. The current study also investigated the effect of the HDAB-induced S phase arrest on the proliferation and apoptosis of cervical cancer cells. Unexpectedly, the attenuation of S phase arrest by cyclin A2 knockdown or CDK2 inhibition further enhanced the HDAB-induced apoptosis and colony formation inhibition. These results indicated that the activation of cyclin A/CDK2 and S phase arrest are not the real causes of the HDAB-induced apoptosis.

DNA damage agents can activate DDR pathway and cause cell cycle arrest to enable DNA repair in several human cancer cell lines^19^,^20^,^21^,^22^. Our data showed that HDAB elicited DNA damage (Fig. 5) and activated the ATM-dependent DNA repair pathway, but not the DNA-PK pathway, in cervical cancer cells (Fig. 6). ATM can also induce pro-apoptotic pathways in case of irreparable damage or damage overload. In this study, low concentrations of HDAB initiates the cell programmed death of cervical cancer cells, indicating that HDAB might result in persistent double strand breaks and therefore ATM elicits a strong apoptotic signal. In addition, it is a very well established fact that ATM inhibition leads to sensitivity to DNA damage, most notably to double strand breaks. Consistent with this hypothesis, the inhibition of ATM activation by the ATM-specific inhibitor CGK733, but not the DNA-PK-specific inhibitor NU7026, effectively abolished the HDAB-induced S phase arrest and enhanced apoptosis and colony formation inhibition in cervical cancer cells (Fig. 7).

**Figure 9:**
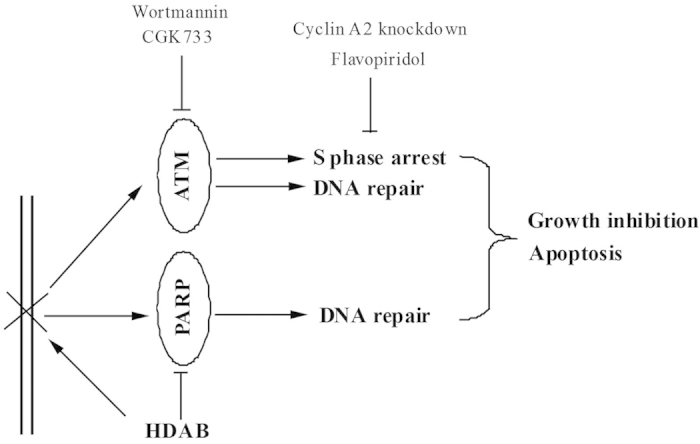
The possible signal pathways regulated by HDAB in cervical cancer cells. HDAB treatment activates the ATM-dependent DNA damage response and induces S phase arrest. An ATM kinase inhibitor, a CDK2 inhibitor and cyclin A2 siRNA can significantly increase the HDAB-induced apoptosis of cervical cancer cells. In addition, HDAB can function as an inhibitor of PARP to impair DNA repair, thereby enhancing cell death.

Recent studies have shown that PARP plays an important role in the DNA damage repair response, and PARP inhibitors have been regarded as a novel class of anti-cancer agents^41^,^42^,^43^. Once PARP is activated by damaged DNA, it binds to damaged DNA fragments and catalyses the cleavage of NAD^+^into nicotinamide and ADP-ribose to form ADP-ribose units on target proteins, including histones, topoisomerases and PARP itself, resulting in the recruitment of DNA repair machinery^44^. The vast majority of PARP inhibitors are thought to work by inhibiting PARP enzyme activity as competitive inhibitors of NAD^+^and by preventing the DNA damage repair process^45^, thereby ultimately causing programmed cell death. To achieve better anti-tumour activity, PARP inhibitors have been combined with certain DNA-damaging agents, such as radiotherapy or chemotherapy agents, for the treatment of cancer^46^,^47^,^48^,^49^,^50^. In this study, the molecular docking and *in vitro* PARP-1 activity assay confirmed that HDAB can obviously inhibit PARP-1 enzyme activity, with an IC_50_ of 154.2 μM (Fig. 8). Thus, our data indicated that HDAB might be served as a difunctional anti-cancer drug by inducing DNA damage and inhibiting PARP enzyme activity (Fig. 9).

Previous studies have revealed that ATM knockdown has a synthetic lethal interaction with PARP inhibitors^51^,^52^. Moreover, several cancer cell lines that lack ATM function have been shown to have increased sensitivity to PARP inhibitors^53^,^54^. Therefore, we propose that HDAB-mediated PARP inactivation might also have a synergistic effect with ATM inhibitor on the accumulation of HDAB-induced DNA damage, thereby enhancing cell death (Fig. 9). However, we also noticed that the inhibitory ability of HDAB for PARP1 activity was still less potent than the known PARP-1 inhibitors (Fig. 8C). Therefore, the ability of HDAB to inhibit PARP1 also needs to be further improved through chemical modification in the future research.

In summary, we found that the novel natural product HDAB can significantly suppress cervical cancer cell growth and proliferation and induce apoptosis by causing DNA damage and by inhibiting PARP activity. In addition, our study also suggested the possibility of combining targeted agents such as ATM inhibitors and HDAB to induce a synergistic lethal response for the treatment of cervical cancer (Fig. 9).

## Methods

### Cell lines and reagents

7-Hydroxy-5, 4’-dimethoxy-2-arylbenzofuran (HDAB) was isolated and identified as described in our previous study^13^. HDAB was dissolved in dimethyl sulfoxide (DMSO) and stored at −20 °C. The HeLa and CaSki human cervical cancer cell lines, the HL-60 human myeloid leukaemia cell line, the Jurkat human T cell lymphoblast-like cell line, the HepG2 human liver cancer cell line, the CNE-1 human nasopharyngeal carcinoma cell line, MCF-7 (BRCA1 proficient human breast cancer cell line) and MDA-MB-436 (BRCA1 deficient human breast cancer cell line) were purchased from the American Type Culture Collection (ATCC). The cells were cultured in DMEM or RPMI 1640 medium containing 10% foetal bovine serum (FBS) under standard culture conditions (95% humidified air and 5% CO_2_ at 37 °C). Primary antibodies against Phospho-ATM (Ser1981), ATM, phospho-CHK1 (Ser345), phospho-CHK2 (Thr68), CHK1, CHK2, cyclin D1, Phospho-Histone H2A.X (Ser139), Cleaved Caspase-3 (Asp175), and Phospho-(Ser/Thr) ATM/ATR substrates were purchased from Cell Signalling (Beverly, MA, USA). The primary antibodies against cyclin E, cyclin A2, CDK2, and p21 were purchased from Abcam (Abcam, Cambridge, UK). The phospho-DNA-PKCS (Thr 2609) and DNA-PK antibodies were purchased from Santa Cruz Biotechnology (Santa Cruz, CA, USA). HRP- and fluorescein-labelled secondary antibodies and the ECL detection system were obtained from KPL (Gaithersburg, MD, USA). Wortmannin, LY294002, NU7026, and CGK733 were obtained from Sigma-Aldrich (St. Louis, MO, USA). Propidium iodide (PI) and 3-(4,5-dimethylthiazol-2-yl)-2,5-diphenyltetrazolium bromide (MTT) were purchased from Sigma-Aldrich (St. Louis, MO, USA). A mitochondrial membrane potential assay kit with JC-1 was purchased from Beyotime (Nanjing, Jiangsu, China). An Alexa® Fluor 488 Annexin V/PI Dead Cell Apoptosis Kit was obtained from Invitrogen (Carlsbad, CA, USA). Previously reported chemically synthesized control and cyclin A2 siRNAs were purchased from GenePharma (GenePharma, Shanghai, China)^55^. The cyclin A2 siRNA sequences are as follows: siRNA1, 5’-CCAUUGGUCCCUCUUGAUUTT-3’; siRNA2, 5’-CAGGACCAGGAGAAUAUCATT-3’. The nonsilencing control siRNA sequence is 5’-UUCUCCGAACGUGUCACGUTT-3’.

### Cell viablility assays by MTT

Cell viability was measured by the MTT assay. Briefly, the cells were plated in a 96-well plate (4 × 10^3^ cells/well). After 24 h, the cells were treated with DMSO or different concentrations of HDAB. After 48 h of treatment, 100 μL of MTT (5 mg/mL in DMEM or RPMI 1640) was added to each well for 4 h, the medium was replaced with 200 μL of DMSO, and the cells were incubated at room temperature in the dark for 6 h. The OD value was measured using a spectrophotometric microtiter plate reader at 570 nm. The IC_50_ of HDAB in the different cell lines was calculated.

### Cell proliferation assay

Cells were seeded at low density (0.2 × 10^6^ cells/10 cm plate). Cell numbers were quantified every day and the data from three independent studies were presented as mean ±standard deviation (SD).

### Colony formation assay

The colony formation ability of the cervical cancer cells or human breast cancer cells was evaluated by soft agar colony formation assay. Briefly, the base agar was prepared by mixing 2 × medium, 1.2% agar, 20% FBS and antibiotics. Then, the top agar was prepared by mixing 2 × medium, 0.6% agar, 20% FBS, 2 × antibiotics, HDAB, kinase inhibitors and the same number of cells. The cells were incubated for 10 days. The resulting colonies were stained with 0.1% crystal violet for 30 min and counted by microscopy.

### Apoptosis analysis by annexin V/PI staining

Cells were treated with either DMSO or different concentrations of HDAB in the absence or presence of kinase inhibitors for 24 h. Thereafter, the cells were trypsinised with EDTA-free trypsin and were stained with annexin V and PI according to the manufacturer’s instructions for the Dead Cell Apoptosis Kit (Invitrogen, V13241). Apoptosis was detected by flow cytometry. Annexin V-positive cells were considered apoptotic cells.

### Apoptosis analysis by DAPI staining

After the cells were treated for 24 h with either DMSO or different concentrations of HDAB, they were fixed in pre-chilled methanol for 2 min and then stained with 0.5 μg/mL of 4’, 6-diamidino-2-phenylindole (DAPI) for 10 min. Nuclei were examined and imaged using a fluorescence microscope.

### Mitochondrial membrane potential assay

Cells were treated with DMSO or HDAB. At the desired time point, JC-1 staining solution was added into the culture medium (5 μg/mL) for 15 min. The cells were analysed by confocal microscopy with the following fluorescence design: excitation/emission=540/570 nm for red J-aggregates and excitation/emission =485/535 nm for green monomers.

### Cell cycle analysis

Cells were treated with either DMSO or different concentrations of HDAB in the absence or presence of kinase inhibitors for 24 h. Thereafter, the cells were trypsinised and resuspended in staining buffer (0.3% saponin, 25 mg/mL PI, 0.1 mM EDTA and 10 mg/mL RNase in PBS) at 4 °C for 24 h. Then, the cell cycle distribution was analysed by flow cytometry.

### Immunoblot analysis

Cells were treated with HDAB in the absence or presence of various kinase inhibitors and then harvested and lysed in cell lysis buffer (1% SDS, 10 mM EDTA, 50 mM Tris-HCl (pH 8.0) and 0.1 mM PMSF) at the appropriate time points. Equal amounts of protein were subjected to SDS-PAGE and then transferred onto PVDF membranes. The membranes were blocked with 5% non-fat milk for 2 h at room temperature and then incubated with the desired primary antibodies overnight at 4 °C. Subsequently, the proteins were detected using ECL reagents after a 4 h incubation at room temperature with HRP-labelled secondary antibodies.

### Comet assay

Cells were treated with HDAB for 24 h, and single-cell suspensions were prepared by mixing 1 × 10^6^ cells with 1.5 mL of 1% agarose. The cell suspension was layered onto a glass microscope slide, and then the slide was placed in lysis buffer (1% N-lauroylsarcosine, 1M NaCl, 1 mM EDTA, 10 mM Tris-HCl, 30 mM NaOH, 1% Triton X-100 and 10% DMSO, pH 10.0) for 1 h at 4 °C. Electrophoresis was performed at 0.6 V/cm in electrophoresis buffer (30 mM NaOH and 1 mM EDTA, pH 10.0) for 20 min. Thereafter, the slide was neutralised with neutralisation buffer (0.4 M Tris-HCl, pH 7.5) and stained with propidium iodide (2.5 μg/mL) for 10 min. The DNA damage levels were analysed, and the slides were imaged using a fluorescence microscope.

### Immunocytochemical staining for γH2A.X (Ser139)

Cells were seeded in a 12-well plate containing cover glasses one day before HDAB treatment. DMSO alone or different concentrations of HDAB with or without kinase inhibitors were added to the medium. At the end of the desired treatment times, the cells on the cover glasses were fixed in 4% paraformaldehyde for 15 min and permeabilised with 0.1% Triton X-100 for 5 min. After the cells were sequentially incubated with primary antibody against γH2A.X (Ser139), fluoresein-conjugated secondary antibody and DAPI, they were observed by confocal microscopy.

### Nude mouse xenograft model

Six-week-old female nude mice (Balb/c-nu/nu) were purchased from the Medical Experimental Animal Centre of Guangdong Province. The animal experiments were approved by the Institutional Animal Care and Use Committee of Tsinghua University. Mice were randomly allocated into 2 groups with 6 animals per group. Exponentially growing HeLa cells (2 × 10^6^ cells in 100 μL) were injected subcutaneously into the backs of the mice. When tumours become apparent, DMSO or HDAB was administered *via* intraperitoneal injection into tumour-bearing mice in the control group or treatment group, respectively, every 2 days at a single dose of 1 mg per mouse. The lengths and widths of the tumours were measured using a vernier calliper, and the tumour volumes were calculated using the following equation: Volume = (length×width^2^) / 2. The mice were sacrificed on day 20 post-HDAB treatment, and the tumour tissues were collected, weighed, and imaged.

All of the animals were treated according to protocols approved by the Institutional Animal Care and Use Committee of Tsinghua University. And this study was approved by the Institutional Animal Care and Use Committee of Tsinghua University.

### PARP-1 activity assay

PARP activity was monitored using an HT Universal Colorimetric PARP Assay Kit (Cat# 4677-096-K) from Trevigen (Gaithersburg, MD, USA). Briefly, the PARP-1 inhibitor 3-aminobenzamide (3-AB) and HDAB were serially diluted, and their ability to inhibit PARP-1 was measured according to the manufacturer’s protocol. The results are presented as the percent inhibition compared to the control wells.

### Statistical analysis

The results are presented as the mean ±standard deviation (SD) calculated from three independent experiments. Student’s *t*-test was used to compare the differences in all the measurable variables in this study. Differences with *p* < 0.05 were considered significant.

## Additional Information

**How to cite this article**: Chen, H. *et al.* A new arylbenzofuran derivative functions as an anti-tumor agent by inducing DNA damage and inhibiting PARP activity. *Sci. Rep.* **5**, 10893; doi: 10.1038/srep10893 (2015).

## Figures and Tables

**Figure 1:**
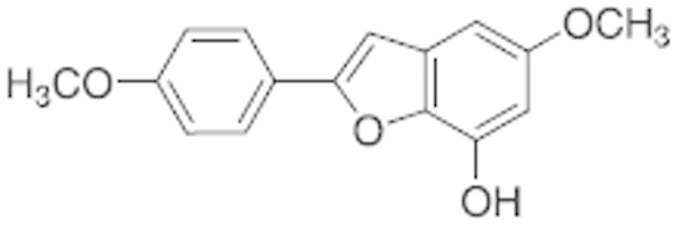
Chemical structure of 7-hydroxy-5,4’-dimethoxy-2-arylbenzofuran.

**Table 1:** Antiproliferative activities of HDAB against several human cancer cell lines.

**Cell line**	**HL-60**	**Jurkat**	**HepG2**	**CNE-1**	**HeLa**	**CaSki**
IC_50_ (24 hμM)	18.44 ± 0.85	29.89 ± 1.52	28.15 ± 1.74	16.63 ± 0.15	11.07 ± 0.44	11.41 ± 0.33
IC_50_ (48h μM)	11.29 ± 0.11	17.55 ± 1.78	14.37 ± 0.26	9.52 ± 0.11	5.52 ± 0.26	6.55 ± 1.22
